# Rectal dose to prostate cancer patients treated with proton therapy with or without rectal spacer

**DOI:** 10.1002/acm2.12001

**Published:** 2016-11-21

**Authors:** Heeteak Chung, Jerimy Polf, Shahed Badiyan, Matthew Biagioli, Daniel Fernandez, Kujtim Latifi, Richard Wilder, Minesh Mehta, Michael Chuong

**Affiliations:** ^1^ Department of Radiation Oncology University of Maryland Baltimore School of Medicine Baltimore MD USA; ^2^ Department of Radiation Oncology Florida Hospital Cancer Institute Orlando FL USA; ^3^ Department of Radiation Oncology H. Lee Moffitt Cancer Center Tampa FL USA

**Keywords:** proton beam scanning, prostate cancer, rectal spacers

## Abstract

The purpose of this study was to evaluate whether a spacer inserted in the prerectal space could reduce modeled rectal dose and toxicity rates for patients with prostate cancer treated *in silico* with pencil beam scanning (PBS) proton therapy. A total of 20 patients were included in this study who received photon therapy (12 with rectal spacer (DuraSeal™ gel) and 8 without). Two PBS treatment plans were retrospectively created for each patient using the following beam arrangements: (1) lateral‐opposed (LAT) fields and (2) left and right anterior oblique (LAO/RAO) fields. Dose volume histograms (DVH) were generated for the prostate, rectum, bladder, and right and left femoral heads. The normal tissue complication probability (NTCP) for ≥grade 2 rectal toxicity was calculated using the Lyman–Kutcher–Burman model and compared between patients with and without the rectal spacer. A significantly lower mean rectal DVH was achieved in patients with rectal spacer compared to those without. For LAT plans, the mean rectal V70 with and without rectal spacer was 4.19 and 13.5%, respectively. For LAO/RAO plans, the mean rectal V70 with and without rectal spacer was 5.07 and 13.5%, respectively. No significant differences were found in any rectal dosimetric parameters between the LAT and the LAO/RAO plans generated with the rectal spacers. We found that ≥ 9 mm space resulted in a significant decrease in NTCP modeled for ≥grade 2 rectal toxicity. Rectal spacers can significantly decrease modeled rectal dose and predicted ≥grade 2 rectal toxicity in prostate cancer patients treated *in silico* with PBS. A minimum of 9 mm separation between the prostate and anterior rectal wall yields the largest benefit.

## 1 Introduction

Dose‐escalated external beam radiation therapy (EBRT) (≥ 78 Gy) has replaced the previous standard dose of ~70 Gy as the new “standard of care” for the treatment of prostate cancer.^1^, ^2^, ^3^, ^4^, ^5^, ^6^, ^7^, ^8^, ^9^, ^10^, ^11^, ^12^ Dose‐escalated EBRT significantly improves biochemical progression‐free survival in most patients, and also overall survival in patients with intermediate‐ or high‐risk disease.^7^, ^13^, ^14^ However, escalating the dose to the prostate has also led to an increase in ≥grade 2 rectal toxicity rates.^15^, ^16^, ^17^ Grade ≥ 2 rectal toxicity has been shown to be significantly associated with the volume of rectum receiving > 70 Gy (V70).^14^ Thus, techniques to minimize the rectal V70 are likely to translate into clinically meaningful benefits.

In order to maximize the therapeutic benefit of dose escalation to the prostate, the use of a tissue spacer to increase the physical distance between the prostate and the rectum has been studied^18^, ^19^, ^20^, ^21^, ^22^ in order to minimize rectal toxicities. Recently, Mariados et al.^18^ reported a significant reduction in the volume of the rectum receiving 70 Gy or more (V70) from 12.4 to 3.3% (*P* < 0.01), for patients treated with image‐guided intensity modulated radiation therapy (IMRT), planned pre‐ and postspacer.

Proton therapy has advantageous dose deposition properties resulting in a decrease in the volume of normal tissues irradiated compared to photon irradiation. Proton therapy has been delivered in the past using a passive system (passive scattering), but a more conformal and highly modulated beam can be created using actively scanned proton beamlets (PBS) that are overlaid over the target volume.^23^ Proton therapy has become an increasingly utilized treatment for patients with prostate cancer,^24^, ^25^, ^26^, ^27^ and a large number of PBS proton therapy centers are scheduled to open in the next 5 yr.

Recent retrospective reviews have cast doubt on the value of proton therapy for the treatment of prostate cancer, with some studies suggesting low rates of rectal toxicity compared to IMRT, while other suggesting similar rates.^28^, ^29^ PBS proton therapy may prove to result in superior quality of life in the long run, but currently questions remain regarding its use relative to rectal toxicity.

The purpose of this study was to evaluate whether the use of a rectal spacer could reduce modeled rectal toxicity for patient undergoing *in silico* pencil beam scanning (PBS) proton therapy for prostate cancer, to quantify the modeled differences, and to evaluate the minimum space requirement between the prostate and rectum to yield a significant reduction in V70.

## 2 Methods

### 2.1 Rectal spacer

Twelve nonmetastatic prostate cancer patients treated with definitive radiotherapy were included in this IRB‐approved study. Each had DuraSeal™ gel (Covidien, Mansfield, MA, USA) inserted percutaneously through the peritoneum posterior to Denonvillier's fascia and anterior to the rectum. An ultrasound image‐guided injection of the gel (5 ml) via 16‐guage catheter was performed to the level of the mid prostate gland to slowly separate the anterior perirectal fat, thus creating a space for the gel at the level of the mid prostate gland.^30^ The injection of DuraSeal™ gel was done prior to CT simulation by a radiation oncologist with significant brachytherapy experience (M.B. or D.F.). In this study, we refer to the DuraSeal™ gel as the rectal spacer.

The separation distance between the rectum and the prostate was measured for all patients. The measurements were taken at three points; superior, middle, and inferior aspects of the prostate gland on an axial plane using the planning CT images. The minimum separation distance in any of these three measurements was used for our analysis regarding the impact of separation distance on reduction in modeled toxicities.

### 2.2 Proton planning

The CT scans for each of the 12 patients (Patients #1–12) with the rectal spacer in place were utilized to retrospectively generate PBS treatment plans using the Varian Eclipse treatment planning system (Version 13; Varian Medical Systems Inc., Palo Alto, CA, USA). To perform dosimetric evaluations for patients with and without rectal spacers for PBS plans, a control group of eight randomly selected prostate cancer patients (Patients #13–20) who received definitive IMRT without a rectal spacer also had PBS proton treatment plans. For each plan, the prostate was contoured on the planning CT scan and labeled as the clinical target volume (CTV). The CTV was expanded 6 mm isotropically to create the planning target volume (PTV). Additionally, the rectum, bladder, and femoral heads were contoured for all patients. The volumes of the prostate, PTV, rectum, and bladder for all patients are listed in Table 1. The total dose prescribed to the PTV was 79.2 Gy in 44 fractions, defined as at least 95% of the PTV receiving 100% of the prescribed dose. Dose‐volume constraints to the organs at risk were applied as follows: For the bladder (full bladder), the volume receiving 80 Gy or more (V80) was limited to ≤ 15%, V75 ≤ 25%, V70 ≤ 35%, and V65 ≤ 50%. For the rectum (empty rectum), constraints of V75 ≤ 15%, V70 ≤ 25%, V65 ≤ 35%, and V60 ≤ 50% were used. The maximum femoral head point dose could not exceed 50 Gy.

**Table 1:** Volume of targets and critical organs

Patient #	Volume (cm^3^)			
	PTV	CTV (Prostate)	Rectum	Bladder
With rectal spacer				
1	124.6	52.5	47.6	119.8
2	105.8	39.4	62.4	116.9
3	110.1	45.7	60.6	99.8
4	114	53	35.9	120.4
5	124.6	52.5	47.6	119.8
6	169.3	75.1	84.7	190
7	185.3	91.2	62	250.3
8	147	64.4	47.1	80
9	134.7	62.9	38.4	141.4
10	153.7	61.8	91.2	69.4
11	87.6	33.5	108.9	136.2
12	138	61	48.1	293.5
Without rectal spacer				
13	58.9	17.2	31.5	147.3
14	226	91	89.8	223.9
15	145	63.8	95.8	71.3
16	94.5	44	42.4	98.4
17	181.9	40.5	135.3	168.6
18	276	156.9	129.3	252.9
19	96	40.2	118.8	425.1
20	78.9	27.7	48.6	197.5
Mean	137.595	58.715	71.3	166.125
Median	129.65	52.75	61.3	138.8

Conventionally, anterior fields are avoided and only lateral fields are used when treating the prostate with proton therapy because of range uncertainty considerations. However, anterior–posterior proton beams have the potential to better spare the anterior rectal wall, and the presence of the spacer mitigates the range uncertainty concern of having the highest post‐Bragg peak RBE component of the proton beam hitting the rectal wall. Therefore, we created two proton plans for each patient with the spacer: (1) lateral‐opposed (LAT) fields (90° and 270° gantry angles), and (2) two anterior oblique (LAO/RAO) fields (60° and 300° gantry angles). The coordinate systems are defined according to IEC 61217. The plans were optimized using equally weighted single field optimization (SFO). The proximal and distal margins of each field were determined from the water equivalent thickness (WET) of the proximal and distal edge of the PTV. Additionally, to account for beam range uncertainty,^31^ an additional 3.5% of proximal and distal WET plus 2 mm (3.5%·WET_Proximal/Distal_ + 2 mm) was added to the PTV.

### 2.3 Dose volume histogram analysis

Dose volume histograms (DVH) were calculated for the CTV, rectum, bladder, and right and left femoral heads for both beam arrangements (LAT and LAO/RAO) for all patients. For the CTV, the D100 and dose maximum (Dmax) were evaluated. Evaluation was performed for all planning goals and constraints listed above for the CTV, rectum, bladder, and femoral heads. For this study, no robustness evaluation was performed.

### 2.4 Rectal NTCP

Lyman–Kutcher–Burman (LKB) methodology was used to determine the predicted rectal toxicity rates for each beam arrangement.^32^ The LKB model describes the normal tissue complication probability (NTCP) after uniform radiation of a fractional volume (*v*) of normal tissue to a dose (*D*) using the equation:

(1) $$NTCP=\frac{1}{2\sqrt{\pi }}\int_{-\infty }^{t}e^{-\frac{x^{2}}{2}}dx,$$

where

(2) $$t=\frac{gEUD-TD_{50}}{m\cdot TD_{50}},$$

*TD*_*50*_ is the dose at which there is a 50% probability of developing a specified grade and type of rectal complication after uniform whole‐organ irradiation, *m* models the slope of the dose–response curve for this specific toxicity. The term *gEUD* is the generalized equivalent uniform dose which accounts for the fact that the rectum does not receive a uniform dose during treatment and is calculated according to the Kutcher–Burman histogram dose reduction method,^32^

(3) $$gEUD={\left[\frac{1}{N_{voxels}}\sum_{i=1}^{N_{voxels}}D_{i}^{1/n}\right]}^{n},$$

where *N*_*voxels*_ is the number of voxels, *D*_*i*_ is the dose to the i_th_ voxel, and *n* is the volume effect factor, which models how the tolerance dose changes as the fractional volume of the rectum irradiated changes. To evaluate a minimum of grade 2 rectal toxicity, QUANTEC‐recommended parameters were used.^33^ They are as follows: *n* = 0.09 (0.04 – 0.14), *m* = 0.13 (0.1 – 0.17), and *TD*_*50*_ = 76.9 Gy (73.7 – 80.1).

## 3 Results

### 3.1 Proton treatment plan evaluation

For both the LAT and LAO/RAO field arrangements, target volume coverage and critical structure planning goals were achieved for all patients regardless of the beam arrangements or the presence of a rectal spacer.

### 3.2 DVH comparison

Table 2 shows tabulated mean and standard deviation (1*σ*) DVH values for LAT and LAO/RAO field arrangements for patients with and without rectal spacers. Table 2 lists mean DVH values for CTV, rectum, bladder, and right and left femoral heads. For the LAT field arrangement, the mean values for D100 for CTV for rectal spacer and no rectal spacer patients were 79.77 and 79.48 Gy, respectively. The CTV Dmax with and without rectal spacer was 81.77 and 81.67 Gy, respectively. The rectal mean Dmax with and without rectal spacer was 77.06 and 81.45 Gy, respectively. A significant difference was seen between the mean values for all relevant QUANTEC dose volume metrics favoring patients with a rectal spacer (*P* < 0.05). The bladder mean Dmax with and without rectal spacer was 81.60 and 81.56 Gy, respectively. For the bladder QUANTEC dose volume metrics, only the V80 showed significant difference with and without rectal spacer (*P* = 0.03). The right femoral head mean Dmax with and without rectal spacer was 37.25 and 34.06 Gy, respectively. The left femoral head mean Dmax with and without rectal spacer was 35.93 and 34.24 Gy, respectively. For both femoral heads, no QUANTEC dose volume metrics showed any significant difference with and without rectal spacer.

**Table 2:** Mean DVH values for LAT and LAO/RAO fields

			CTV		Rectum					Bladder					RT Femoral Head			LT Femoral Head		
			D100 (Gy)	Dmax (Gy)	Dmax (Gy)	V60 (%)	V65 (%)	V70 (%)	V75 (%)	Dmax (Gy)	V65 (%)	V70 (%)	V75 (%)	V80 (%)	Dmax (Gy)	V15 (%)	V30 (%)	Dmax (Gy)	V15 (%)	V30 (%)
LAT	Rectal spacer	Mean	79.77	81.77	77.06	7.37	5.77	4.19	2.52	81.60	13.01	10.77	8.05	3.26	37.25	66.23	33.25	35.93	74.66	35.01
		SD^a^	0.23	0.17	7.39	6.91	6.00	4.95	3.62	0.22	7.78	6.85	5.64	3.49	8.04	16.97	13.64	2.87	7.79	12.29
	No rectal spacer	Mean	79.48	81.67	81.45	18.76	16.01	13.53	9.69	81.56	19.95	17.00	13.66	9.00	34.06	70.53	26.86	34.24	70.58	26.83
		SD^a^	0.23	0.17	0.27	5.23	4.70	4.05	3.41	0.24	11.86	10.61	9.09	6.78	1.02	9.05	16.87	1.77	10.95	16.84
	*P*‐value	0.02	0.25	0.13	<0.01	<0.01	<0.01	<0.01	0.73	0.15	0.15	0.12	0.03	0.30	0.54	0.39	0.17	0.37	0.25
LAO/RAO	Rectal spacer	Mean	79.81	81.79	78.31	9.62	7.32	5.07	2.82	81.74	14.17	11.45	8.26	3.57	34.17	16.38	2.35	32.69	13.21	1.56
		SD^a^	0.17	0.40	4.92	8.09	6.99	5.65	3.86	0.47	7.47	6.33	4.98	3.12	6.35	4.34	2.30	2.72	5.45	1.37
	No rectal spacer	Mean	79.42	81.49	81.42	19.32	16.53	13.53	10.36	81.69	21.41	18.03	14.49	9.55	32.67	17.32	3.85	32.55	15.15	3.20
		SD^a^	0.19	0.16	0.29	5.85	5.03	4.31	3.20	0.66	12.67	10.76	9.21	6.78	2.75	9.05	4.33	3.35	8.10	4.13
	*P*‐value	<0.01	0.07	0.11	0.01	0.01	<0.01	<0.01	0.85	0.15	0.12	0.08	0.02	0.56	0.77	0.35	0.93	0.55	0.24	

a 1 standard deviation.

For the LAO/RAO plans, the mean values for D100 for CTV with and without the rectal spacer patients were 79.81 and 79.42 Gy, respectively. The CTV Dmax values for patients with rectal spacer compared to no spacer were 81.79 and 81.49 Gy, respectively. The mean rectal Dmax for patients with rectal spacer compared to those without was 78.31 and 81.42 Gy, respectively. A significant difference was observed between the mean values for all relevant QUANTEC dose volume metrics for the rectal spacer patients compared to those without (*P* < 0.05). For the bladder, the mean Dmax with and without the rectal spacer was 81.74 and 81.69 Gy, respectively. The only significant difference in favor of the rectal spacer was found for the V80 (*P* = 0.02). The right femoral head mean Dmax with and without rectal spacer was 34.17 and 32.67 Gy, respectively. The left femoral head mean Dmax with and without rectal spacer was 32.69 and 32.55 Gy, respectively. For both femoral heads, no QUANTEC dose volume metrics showed any significant difference with and without rectal spacer. Furthermore, there was no significant difference in rectal or bladder dose when using the rectal spacer between the LAT and RAO/LAO plans.

**Figure 1:**
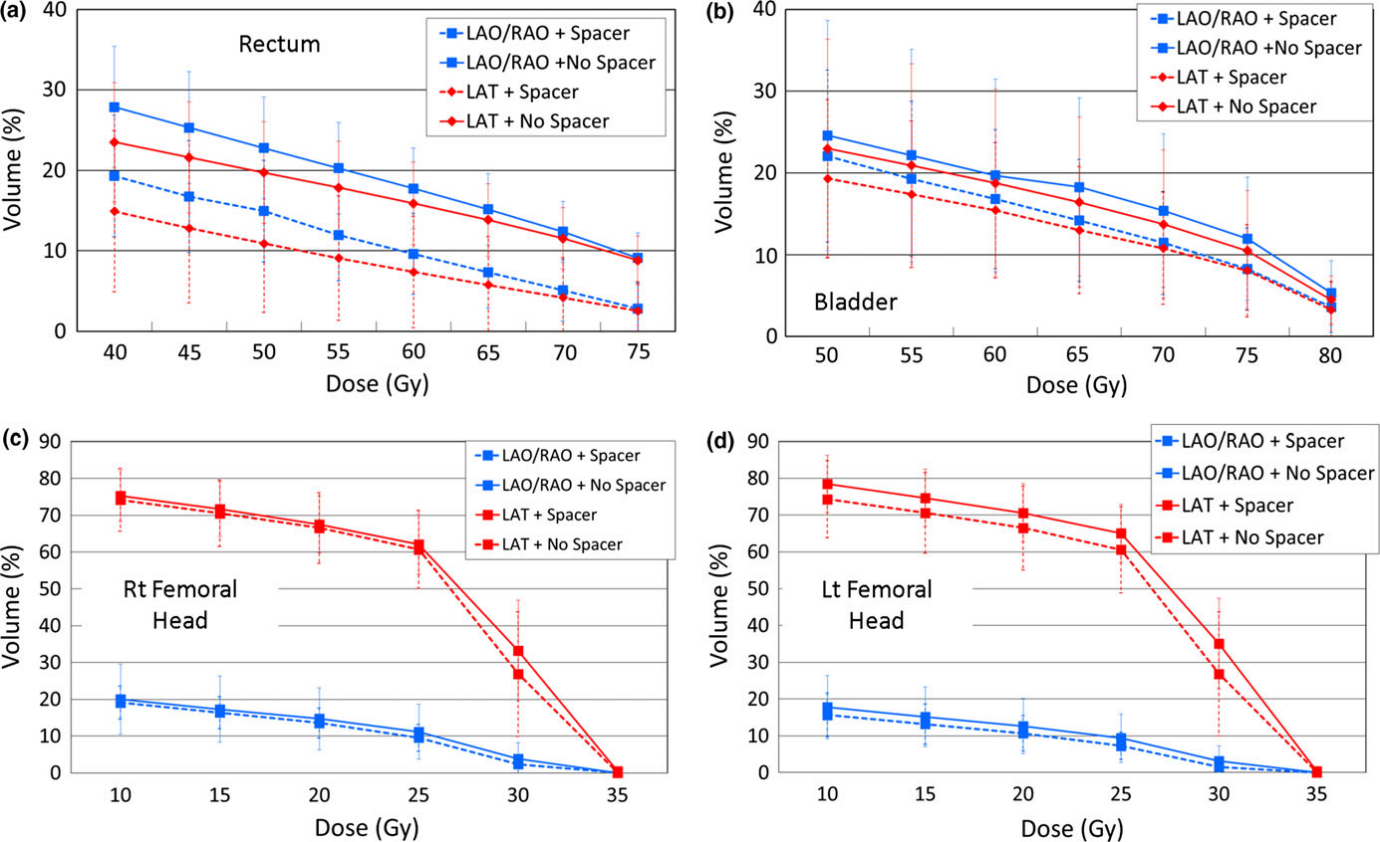
Plots of mean DVH (Gy) versus volume (%) for LAO/RAO and LAT fields with and without rectal spacer. The dotted line represents LAO/RAO and LAT fields with rectal spacer. The error bars indicate +/‐ 1 standard deviation. The solid line represents LAO/RAO and LAT fields without rectal spacer. (a) Rectum, (b) Bladder, and (c) Right Femoral Head and (d) Left Femoral Head.

Population mean rectal and bladder volumes receiving various population mean doses, as a function of the presence/absence of the rectal spacer and beam arrangement, either LAT or LAO/RAO are illustrated in Figs. 1 (a) and 1(b). Figs. 1 (c) and 1(d) are the mean right/left femoral head volumes receiving various mean doses with respect to the presence/absence of the rectal spacer and beam arrangement. The rectal and bladder mean doses are significantly lower for both beam arrangements for the patients with the rectal spacer, and this difference is statistically significant for the rectum, but not for the bladder (Figs. 1(a) and 1(b)). For the mean right/left femoral head doses, there were no statistically difference between the spacer and no spacer for both field arrangements. However, there were significant difference between the LAO/RAO and the LAT field arrangements whether or not the rectal spacers were present.

**Figure 2:**
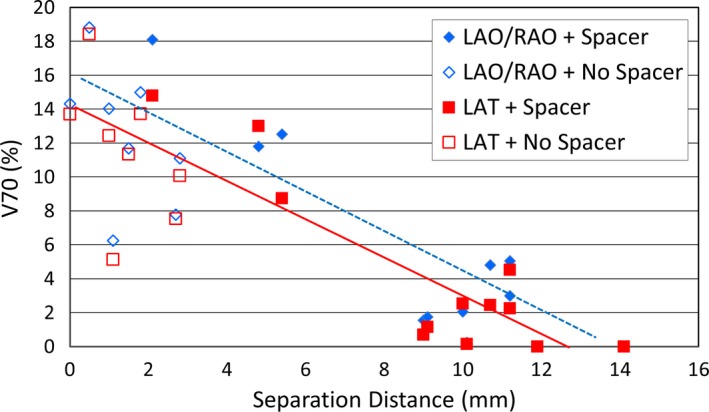
Plot of rectal‐prostate separation distance vs. V70. The filled blue diamond plots are LAO/RAO + Spacer fields. The open blue diamond plots are LAO/RAO + No Spacer fields. The dotted blue line represents the trend for LAO/RAO fields. The filled red square plots are LAT + Spacer fields. The open red square plots are LAT + No Spacer fields. The dotted blue line represents the trend for LAO/RAO fields. The solid red line represents the trend for LAT fields.

The rectum‐prostate separation distance and rectal V70, expressed as % of total contoured rectal volume receiving at least 70 Gy is illustrated in Fig. 2. Both field arrangements show a very similar trend. For patients without a rectal spacer, the rectum‐prostate separation distance ranged from 0 to 2.8 mm with a mean separation distance of 1.4 mm. These patients have V70 values ranging from 5.1% to 18.4%. For patients with rectal spacers, the rectal‐prostate separation distances ranged from 2.1 to 14.1 mm with a mean separation distance of 9.1 mm with the majority of the separation distances clustered between 9 and 12 mm. These patients have V70s ranging from 0 to 18.1% with the majority from 0 to 5%. Only six patients have V70s exceeding 5%, and these six patients have a separation distance of less than 8 mm. Thus, the ideal spacer minimum appears to be at least 9 mm, in order to ensure that the majority of V70 values would fall below 5%.

### 3.3 NTCP analysis

**Figure 3:**
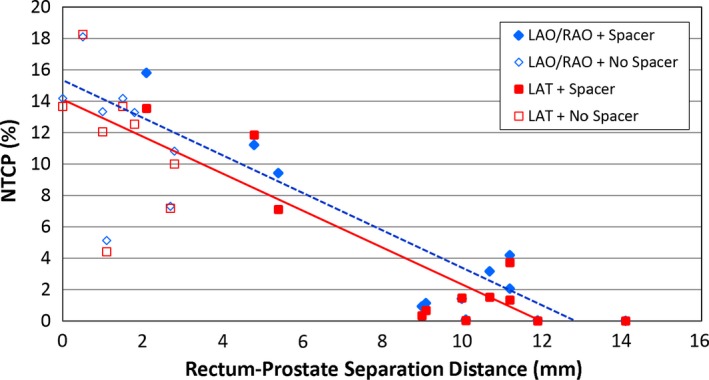
Plot of ≥grade 2 rectal toxicity rates vs. rectum‐prostate separation distance. The filled blue diamond plots are LAO/RAO + Spacer fields. The open blue diamond plots are LAO/RAO + No Spacer fields. The dotted blue line represents the trend for LAO/RAO fields. The filled red square plots are LAT + Spacer fields. The open red square plots are LAT + No Spacer fields. The dotted blue line represents the trend for LAO/RAO fields. The solid red line represents the trend for LAT fields.

The mean predicted rectal toxicity (G2 or higher) probabilities for patients with and without rectal spacers for LAO/RAO and LAT PBS field arrangements are summarized in Table 3. For patients with and without a rectal spacer, the mean predicted rectal toxicity probabilities, expressed as % probability of manifesting ≥grade 2 rectal toxicity for LAO/RAO arrangements were 4.1 and 12.0% (*P* = 0.002), respectively. For LAT beam arrangements, the mean predicted rectal toxicity values for patients with and without rectal spacer were 3.4 and 11.5% (*P* = 0.001), respectively. The patients with the rectal spacers were substantially lower than without the spacers. This represents a 66 and 70% relative reduction in predicted G2 or higher rectal toxicity with the use of spacer, and using either the oblique or the lateral beam arrangements, respectively. The predicted rectal toxicity rates for both field arrangements as a function of the rectum‐prostate separation distance for each patient are illustrated in Fig. 3. For patients without rectal spacers, the NTCP values range from 4.4% to 18.25% %, compared to 0 to 15.8% % for those with rectal spacers, and among these patients, the majority of the NTCP values ranged from 0 to 4.1%. A minimal separation of 9 mm results in the majority of patients having an NTCP risk of 5% or lower, suggesting that such a separation results in V70 of < 5% (Fig. 2) and this corresponds to a < 5% risk of G2 or higher rectal toxicity, an endpoint that could be useful for future clinical trials to validate.

**Table 3:** Mean rates (% probability) of ≥grade 2 rectal toxicity for patients with rectal spacer and without rectal spacer for LAO/RAO and LAT field arrangements

	**LAO/RAO (%)**	**LAT (%)**
Rectal spacer	4.1	3.4
No rectal spacer	12.0	11.5
*P*‐value	0.002	0.001

## 4 Discussion

Our results indicate that a spacer increases the separation between the prostate and rectum, and consistently leads to marked reduction in rectal dose and predicted ≥ grade 2 rectal toxicity (Figs. 2 and 3). This effect was not significantly different based on the use of either LAT or LAO/RAO beam arrangements. These results support those published by Christodouleas et al. who evaluated the dosimetric benefit of using a rectal spacer for prostate proton therapy in a cadaveric study.^34^ In the Christodouleas et al. study, anterior and lateral beam arrangements were compared using uniform scanning and PBS. They reported that the anterior beams improved rectal dosimetric parameters when using uniform scanning, but not when using PBS. Lastly, the data in Fig. 2 suggest a negatively sloped linear trend. In order to reach V70 ≤ 5%, the data suggest the rectum‐prostate separation distance should be approximately 9 mm or greater. Likewise, Christodouleas et al. suggested a similar separation goal (7 mm). Somewhat larger prostate‐rectum separate goals of 10–15 mm have been suggested in the prostate IMRT literature.^35^ Regardless, the published literature demonstrates that at least 7–8 mm of separation can be achieved in most patients.^36^ One important distinction between our study and the one by Christodouleas et al. is that we evaluated the effect of a rectal spacer in a series of patients who received prostate radiotherapy compared to a single cadaver. On that note, our data indicate that despite heterogeneity in spacer placement as well as bladder filling (which can especially affect LAO/RAO plans), the use of a rectal spacer can potentially be beneficial for prostate cancer patients receiving PBS.

Recently, Mariados et al.^18^ reported a prospective randomized rectal spacer study (SpaceOAR) for patients with T1 and T2 prostate cancers undergoing image‐guided IMRT. The mean rectal volumes receiving various mean doses for patients with the rectal spacer were V50 = 12.2% (1σ = ± 8.7%), V60 = 6.8% (1σ = ± 5.5%), and V70 = 3.3% (1σ = ± 3.2%). In our study, the mean rectal volume dose for patients with the rectal spacer for LAO/RAO and LAT were V50 = 14.9% (1σ = ± 10.7%), V60 = 9.6% (1σ = ± 8.1%), and V70 = 5.1% (1σ = ± 5.6%), and V50 = 10.9% (1σ = ± 8.5%), V60 = 7.4% (1σ = ± 6.9%), and V70 = 4.2% (1σ = ± 4.9%), respectively. Based on evaluating the mean rectal volume doses, it can be concluded that the mean rectal volume doses for PBS for the treatment of prostate cancers using rectal spacers are comparable to the IMRT option.

Anterior–posterior‐oriented proton beams have been suggested as potentially useful for treating prostate cancer with PBS, presuming the use of *in vivo* range verification.^37^ However, reliable *in vivo* range verification methods are not currently available for routine use in the clinic. Another disadvantage of using anterior–posterior beams is that they are less robust than lateral beams. Anatomic reproducibility can be challenging for anterior–posterior beams due to the variations in bladder filling. Furthermore, an anterior–posterior beam may not be ideal in overweight men if beams would pass through lower abdominal skin folds, which could be a challenge to reproducibly set up on a daily basis. With the recent Federal Drug Administration (FDA) approval of the first rectal spacer device for prostate cancer patients undergoing radiotherapy (SpaceOAR), we were interested in identifying the potential benefits of anterior‐oriented fields (LAO/RAO). With rectal spacers, we observed significant reductions of V15 and V30 for both femoral heads with anterior‐oriented fields. However, we did not find any difference in the volume of rectum receiving high doses (i.e., V70) for anterior‐oriented fields when compared against the LAT‐oriented fields. This is because the extent of rectal displacement (≥ 9 mm) was significant enough for both field arrangements to observe similar benefits of the rectal spacers to reduce the volume receiving high dose to the rectum. Consequently, while there are some potential benefits of using anterior proton beams *without* a rectal spacer, LAT fields should be preferred *with* a rectal spacer especially because they are also more robust.^25^, ^38^ A rectal spacer may also be especially beneficial for prostate cancer patients with a hip prosthesis who receive proton therapy as an anterior beam is commonly used to avoid treating through the prosthesis, and the LAO/RAO configuration would then be preferred in such situations.^39^

There are several potential limitations of our study. The image dataset obtained for this study did not include CT images before and after the rectal spacer insertion because this study was conceived well after the patients had been treated. However, the study did evaluate the rectal toxicity between the 12 patients with rectal spacers and 8 patients without it. While these eight patients may be the control group (i.e., no rectal spacer), they do represent typical prostate patients for the population comparison. Also, the delineation of tissue interfaces between the rectum, prostate, and rectal spacer was challenging in some patients because of the similarity of their tissue density. To mitigate this, MRI could be used to more clearly delineate the rectal spacer although these patients did not have MRI to guide contouring. Furthermore, there were no patients with rectum‐prostate separation distances between 5 and 9 mm to evaluate potential rectal toxicity in this range. This gap in the rectum‐prostate separation data could influence the determination of minimum spacer thickness needed to provide maximum rectal sparing. Lastly, we did not account for the differences in the day‐to‐day bladder filling that could affect the dose to the rectum for the anterior oblique beams.

## 5 Conclusions

Our modeling study suggests that a rectal spacer can significantly reduce the rectal dose and the probability of ≥grade 2 rectal toxicity among prostate cancer patients who receive PBS. Separation of at least 9 mm between the rectum and prostate may achieve optimal rectal sparing. Opposed lateral beams should be preferred over anterior oblique beams despite the presence of a rectal spacer.
